# Classifying *Oryza sativa* accessions into* Indica* and *Japonica* using logistic regression model with phenotypic data

**DOI:** 10.7717/peerj.7259

**Published:** 2019-11-07

**Authors:** Bongsong Kim

**Affiliations:** Noble Research Institute LLC, Ardmore, OK, Carter, United States of America

**Keywords:** Logistic regression, *Oryza sativa*, *Indica*, *Japonica*, Asian cultivated rice, Genetic diversity in rice, Rice classification, Rice breeding

## Abstract

In *Oryza sativa*, *indica* and *japonica* are pivotal subpopulations, and other subpopulations such as *aus* and *aromatic* are considered to be derived from *indica* or *japonica*. In this regard, *Oryza sativa* accessions are frequently viewed from the *indica/japonica* perspective. This study introduces a computational method for *indica/japonica* classification by applying phenotypic variables to the logistic regression model (LRM). The population used in this study included 413 *Oryza sativa* accessions, of which 280 accessions were *indica* or *japonica*. Out of 24 phenotypic variables, a set of seven phenotypic variables was identified to collectively generate the fully accurate *indica/japonica* separation power of the LRM. The resulting parameters were used to define the customized LRM. Given the 280 *indica/japonica* accessions, the classification accuracy of the customized LRM along with the set of seven phenotypic variables was estimated by 100 iterations of ten-fold cross-validations. As a result, the classification accuracy of 100% was achieved. This suggests that the LRM can be an effective tool to analyze the *indica/japonica* classification with phenotypic variables in *Oryza sativa*.

## Introduction

*Oryza sativa* (Asian cultivated rice) is known to have five subpopulations which are *indica*, *temperate japonica*, *tropical japonica*, *aus* and *aromatic*. Of these, *indica* and *japonica*, comprised of *temperate japonica* and *tropical japonica*, are known as pivotal subpopulations; *aus* is known to be related to *indica*, and *aromatic* is intermediate between *indica* and *japonica* (Garris et al., 2005; Thomson et al., 2007; Kovach et al., 2009; Huang et al., 2012; Schatz et al., 2014; Civan et al., 2015; McCouch et al., 2016; Chin et al., 2017). There are genetic barriers, particularly between *indica* and *japonica,* which often challenge rice trait improvements by breeding (Kato & Kosaka, 1930; Chen et al., 2008; Kim, Jiang & Koh, 2009; Zhu et al., 2017). Because each subpopulation often has desirable characteristics for cultivars, overcoming the genetic barriers between the subpopulations will help rice breeders freely introgress desirable genes originating from different subpopulations into an elite line. This can eventually minimize the breeding costs and maximize the sustainability of rice production being threatened by climate changes, human population increases and loss of cultivation land. Classifying *Oryza sativa* is important because it can help breeders develop effective mating paths to overcome the genetic barriers by identifying accessions that can bridge between *indica* and *japonica*. Currently, classifying *Oryza sativa* is widely conducted with genomic tools because genomic data can reflect the variation of subpopulation characteristics at a molecular level (Garris et al., 2005; Kim, Jiang & Koh, 2009; Zhao et al., 2011; McCouch et al., 2016; Chin et al., 2017). However, the sole use of genomic data may have limitations to the understanding of *Oryza sativa* diversity because some subpopulation-associated traits are related to cytoplasmic effects (Bao, Sun & Corke, 2002; Zhao et al., 2010; Thomson et al., 2007). To overcome the gap that genomic data cannot fill, the use of phenotypic data is reasonable in that it comprehends the genomic and cytoplasmic effects.

This study introduces how to computationally classify *Oryza sativa* accessions into *indica* and *japonica* by applying multiple phenotypic variables to the logistic regression model (LRM). This study used a publicly available data source containing information on 413 *Oryza sativa* accessions and demonstrated that the LRM is a promising tool for accurate *indica*/*japonica* classification in *Oryza sativa* by phenotype.

## Materials and Methods

### Phenotypic data

A set of phenotypic data and subpopulation information was used, which was originally generated and analyzed by Zhao et al. (2011). The data set consisted of 413 accessions originating from 82 countries, obtained at http://ricediversity.org/data/sets/44kgwas. Out of 32 phenotypic variables, 24 variables were selected because they were quantitative and not confined to a certain geography. The selected variables were divided into morphology (culm habit, flag leaf length, flag leaf width), yield components (panicle number per plant, plant height, panicle length, primary panicle branch number, seed number per panicle, florets per panicle, panicle fertility), seed morphology (seed length, seed width, seed volume, seed surface area, brown rice seed length, brown rice seed width, brown rice volume, seed length/width ratio, brown rice length/width ratio), stress tolerance (straighthead susceptibility, blast resistance) and quality (amylose content, alkali spreading value, protein content). Every accession in the data set belonged to one of the following subpopulation groups: *admixed* (62), *aromatic* (14), *aus* (57), *indica* (87), *temperate japonica* (96) and *tropical japonica* (97). In this study, *temperate japonica* and *tropical japonica* were combined into *japonica*.

### Stepwise variable selection and parameter estimation

The LRM formula used in this study can be denoted as follows:

(1) \begin{eqnarray*}P(japonica{|}{x}_{1},\ldots ,{x}_{n})= \frac{1}{1+{e}^{- \left( {\beta }_{0}+{\beta }_{1}{x}_{1}{\cdots +\beta }_{n}{x}_{n} \right) }} \end{eqnarray*}

where *P*(*japonica*|*x*_1_, …, *x*_*n*_) is the probability of an accession being *japonica* (>0.5) or *indica* (<0.5) given the predictor variables, *x*_1_,…, *x*_*n*_; *β*_0_ is the constant term; *β*_1_,…, *β*_*n*_ are the parameters for the predictor variables, *x*_1_, …, *x*_*n*_, respectively.

In Eq. (1), the response variable is binary between *indica* and *japonica*, and the predictor variables (phenotypic variables) are quantitative. To identify a set of predictor variables that can maximize the *indica*/*japonica* separation power, *n* sets were prepared from 1D to *n*D, in which the *n*D is a set containing all possible combinations of *n* different phenotypic variables (e.g., 1D contains every single phenotypic variable, 2D contains all possible pairs of phenotypic variables, and so forth). The *n* was increased by one until the maximum *P*(*japonica*|*x*_1_, …, *x*_*n*_) was reached, in which every single selection from the *n*D set was subject to the following steps:

1. Calculate a set of parameters by fitting the LRM (Eq. (1)) with the phenotypic variables in a selection.
2. Applying the phenotypic variables in the selection used in Step 1 to Eq. (1) with the parameters estimated in Step 1. The resulting value must be between 0 and 1, from which the *indica* or *japonica* can be determined at 0.5.

In order to estimate the *indica*/*japonica* separation power, a receiver operating characteristic (ROC) curve was implemented using an R package called pROC (Robin et al., 2011). In an ROC space, the *x-* and *y-* axes ranging between 0 and 1 represent the false positive rate (FPR or specificity) and true positive rate (TPR or sensitivity), respectively. Thus, an area under an ROC curve (AUC) can range between 0 and 1. The closer the AUC is to 1, the higher the *indica*/*japonica* separation power. By referring to the resulting AUCs, a set of phenotypic variables that maximized the *indica*/*japonica* separation power was identified, and the resulting parameters were used to define the customized LRM.

### Applications of the customized LRM

1. Estimating the *indica*/*japonica* classification accuracy: given the 280 *indica*/*japonica* accessions, the customized LRM along with the related phenotypic variables were taken into 100 iterations of ten-fold cross-validations, from which the 100 values were obtained. The resulting values were averaged into the *indica*/*japonica* classification accuracy.

2. Estimating the variable interaction: as the LRM uses multiple phenotypic variables, some portion of *indica*/*japonica* classification power might be derived from interactions between variables. The interaction magnitude for every variable with all other variables was calculated using the *H*-statistic (Frieman & Popescu, 2008). The resulting *H*-statistic can range between 0 and 1 with no interaction resulting in 0 and full interaction resulting in 1. The *H*-statistic was calculated using an R package, iml (Molnar, Casalicchio & Bischl, 2018).

3. Classifying accessions in each minor subpopulation group into *indica* and *japonica*: the customized LRM was applied to each minor subpopulation group (*aromatic*, *aus*, *admixed*) to divide accessions into *indica* and *japonica*. This examination aimed to observe how accessions in each minor subpopulation group are phenotypically divided from the *indica*/*japonica* perspective.

### Dendrogram-based *indica*/*japonica* classification

To draw dendrograms, the genomic data set was obtained at http://ricediversity.org/data/sets/44kgwas (Zhao et al., 2011). The genomic data set consisted of the accessions in the phenotypic data, genotyped with 36,901 SNPs. Two dendrograms were drawn; one included the 280 *indica*/*japonica* accessions, and the other included all 413 accessions. Then, the dendrogram-based *indica*/*japonica* classifications were compared with the LRM-based *indica*/*japonica* classifications. Each dendrogam was graphed based on a genetic distance matrix in which the genetic distances between two different accessions were calculated using the following equation:

(2) \begin{eqnarray*}Genetic distance between A and B=2-{IBS}_{A,B}\end{eqnarray*}

where the *IBS*_*A*,*B*_ is the IBS (identical by state) coefficient between A and B, which can range between 0 and 2.

The IBS matrix was computed using Numericware i (Kim & Beavis, 2017) which is freely available at https://figshare.com/articles/Numericware_i/3496787.

### Data availability

With the exception of calculating the IBS matrix, all other computations were conducted using R (R Core Team, 2016). The data set and R scripts used in this study are freely available at https://github.com/bongsongkim/logit.regression.rice.

## Results

### Stepwise variable selection

**Figure 1:**
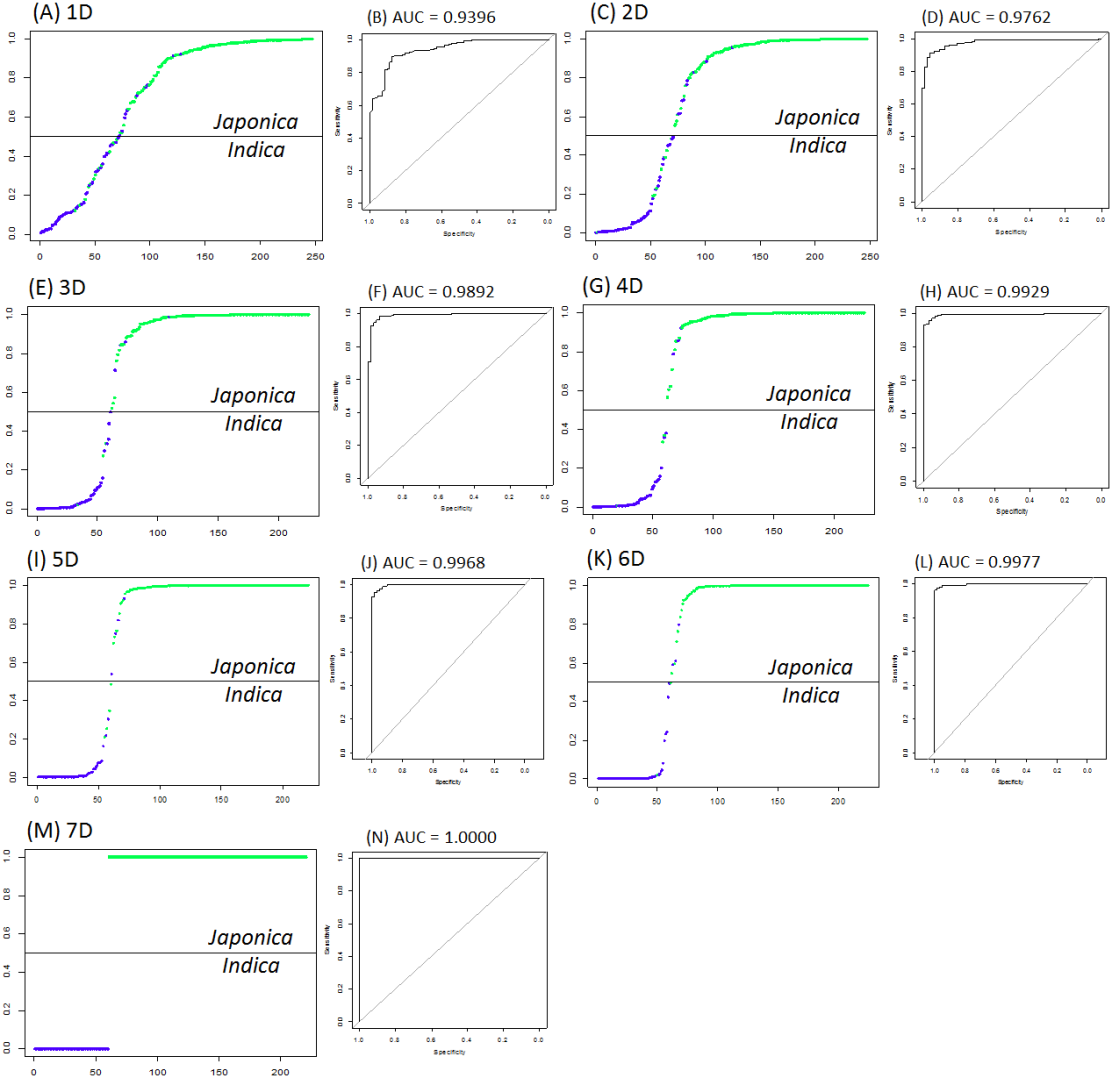
The best indica/japonica classification for each set (1D to 7D). (A) The best *indica/japonica* classification in 1D set, (B) the ROC curve for Fig. 1A, (C) the best *indica/japonica* classification in 2D set, (D) the ROC curve for Fig. 1C, (E) the best *indica/japonica* classification in 3D set, (F) the ROC curve for Fig. 1E, (G) the best *indica/japonica* classification in 4D set, (H) the ROC curve for Fig. 1G, (I) the best *indica/japonica* classification in 5D set, (J) the ROC curve for Fig. 1I, (K) the best *indica/japonica* classification in 6D set, (L) the ROC curve for Fig. 1K, (M) the best *indica/japonica* classification in 7D set, (N) the ROC curve for Fig. 1M.

Figure 1 and Table 1 suggest that more phenotypic variables led to stronger *indica*/*japonica* separation power of the LRM, and the fully accurate *indica*/*japonica* separation power (AUC = 1) was achieved with a 7D selection. Table 2 summarizes the phenotypic variables yielding the maximum *indica*/*japonica* separation power in each set (1D to 7D). The set of phenotypic variables yielding AUC = 1 comprises panicle number per plant, seed number per panicle, florets per panicle, panicle fertility, straighthead susceptibility, blast resistance and protein content.

### Estimating the *indica*/*japonica* classification accuracy

Given the seven phenotypic variables yielding AUC = 1, the *indica*/*japonica* classification accuracy was estimated using the 280 *indica*/*japonica* accessions, for which 100 iterations of ten-fold cross-validations were implemented. As a result, the *indica*/*japonica* classification accuracy of 100% was obtained. This indicates that the seven phenotypes were certainly impactful for the fully accurate *indica*/*japonica* classification. Assuming that some portion of classification power might be derived from interactions between variables, the *H*-statistic was calculated for the purpose of estimating how much each variable generates the classification power in collaboration with other variables. Table 3 summarizes the resulting *H*-statistic values, indicating nearly no interactions between variables.

**Table 1:** Summary of the resulting AUCs obtained in each set (1D to 7D).

**Set name**	**Minimum**	**Median**	**Mean**	**Maximum**
1D	0.5129	0.6937	0.6868	0.9396
2D	0.5192	0.7968	0.7829	0.9762
3D	0.5507	0.8441	0.8413	0.9892
4D	0.5861	0.8789	0.8805	0.9927
5D	0.6101	0.9045	0.9081	0.9968
6D	0.6299	0.9266	0.9283	0.9977
7D	0.6785	0.9435	0.9437	1.0000

**Table 2:** Summary of predictor variables yielding the maximum *indica*/*japonica* separation power in each set (1D to 7D).

**Set name**	**Predictor variables**
1D	panicle number per plant
2D	panicle number per plant, brown rice seed width
3D	panicle number per plant, straighthead susceptibility, blast resistance
4D	panicle number per plant, brown rice volume, straighthead susceptibility, blast resistance
5D	panicle number per plant, brown rice volume, straighthead susceptibility, blast resistance, protein content
6D	panicle number per plant, seed number per panicle, florets per panicle, panicle fertility, straighthead susceptibility, blast resistance
7D	panicle number per plant, seed number per panicle, florets per panicle, panicle fertility, straighthead susceptibility, blast resistance, protein content

**Table 3:** *H*-statistic summary representing how much the *indica*/*japonica* classification power was derived from each predictor variable in collaboration with other predictor variables.

**Phenotypic variable**	***H*-statistic**
Panicle number per plant	3.231598e−15
Seed number per panicle	2.233564e−15
Florets per panicle	4.127335e−16
Panicle fertility	3.167362e−16
Straighthead susceptibility	1.660925e−16
Blast resistance	1.994817e−16
Protein content	1.312777e−16

### Dendrogram-based *indica*/*japonica* classification

**Figure 2:**
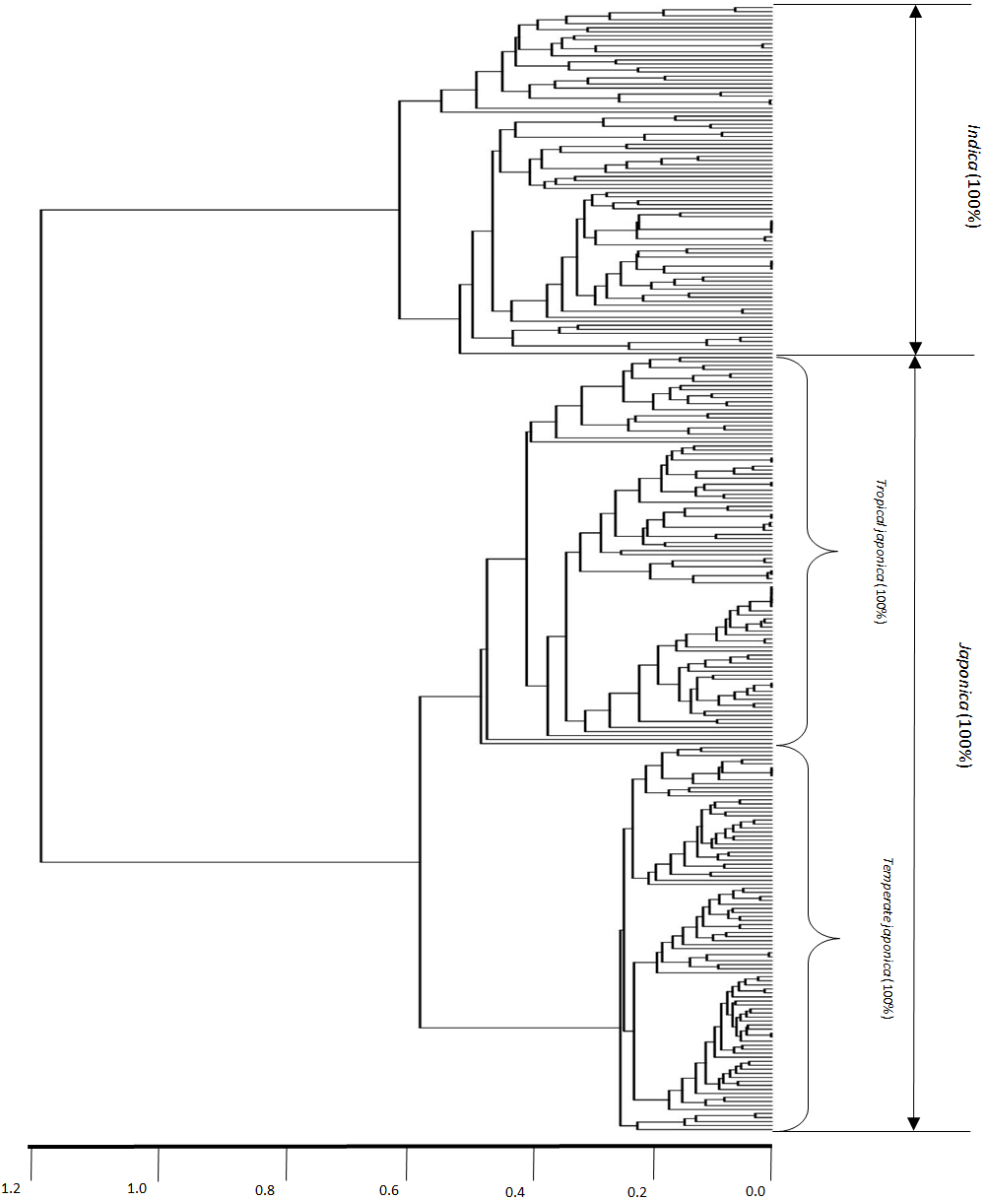
Dendrogram-based *indica/japonica* classification of 280 accessions. Dendrogram-based *indica/japonica* classification of 280 accessions, obtained by using 36,901 SNPs.

Figure 2 shows the dendrogram-based *indica*/*japonica* classification with the 280 *indica*/*japonica* accessions, in which the *indica-* and *japonica-*varietal clades were accurately divided, and the *japonica* accessions were further accurately divided into *temperate japonica* and *tropical japonica* (Fig. S1). This result shows that the dendrogram-based *indica*/*japonica* classification accuracy was 100%.

### Applying the LRM to each minor subpopulation group

The LRM customized with parameters yielding AUC = 1 was used to investigate how it classifies the accessions in each minor subpopulation group (*admixed*, *aromatic*, *aus*) into *indica* and *japonica*. The customized LRM was as follows:

(3) \begin{eqnarray*}P(japonica{|}{x}_{1},\ldots ,{x}_{7})=\nonumber\\\displaystyle \frac{1}{1+{e}^{- \left( 84446.0-5832.5{x}_{1}+35800.4{x}_{2}-38008.7{x}_{3}-50943.2{x}_{4}-491.6{x}_{5}+353.7{x}_{6}-214.6{x}_{7} \right) }} \end{eqnarray*}

where *P*(*japonica*|*x*_1_, …, *x*_7_) is the probability of an accession being *japonica* (>0.5) or *indica* (<0.5) given the predictor variables, *x*_1_,…, *x*_7_; *x*_1_ = the predictor variable for panicle number per plant; *x*_2_ = the predictor variable for seed number per panicle; *x*_3_ = the predictor variable for florets per panicle; *x*_4_ = the predictor variable for panicle fertility; *x*_5_ = the predictor variable for straighthead susceptibility; *x*_6_ = the predictor variable for blast resistance; *x*_7_ = the predictor variable for protein content.

The absolute values for parameters in Eq. (3) in descending order are 50943.2 (-) for panicle fertility, 38008.7 (-) for florets per panicle, 35800.4 (+) for seed number per panicle, 5832.5 (-) for panicle number per plant, 491.6 (-) for straighthead susceptibility, 353.7 (+) for blast resistance and 214.6 (-) for protein content. These suggest that, when it comes to the *indica*/*japonica* separation power, the panicle fertility is most impactful, followed by florets per panicle, seed number per panicle, panicle number per plant, straighthead susceptibility, blast resistance and protein content.

**Figure 3:**
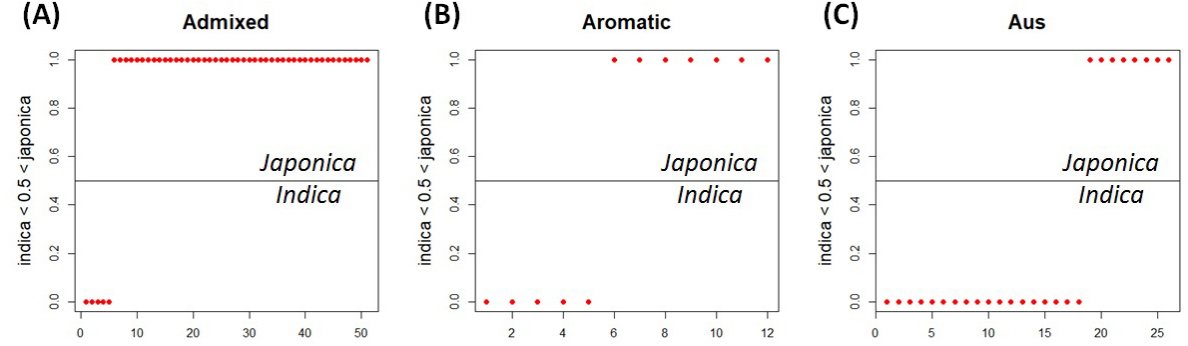
LRM-based *indica/japonica* classification for each minor subpopulation group. (A) LRM-based *indica/japonica* classification for the *admixed* group, (B) LRM-based *indica/japonica* classification for the *aromatic* group, (C) LRM-based *indica/japonica* classification for the *aus* group.

**Figure 4:**
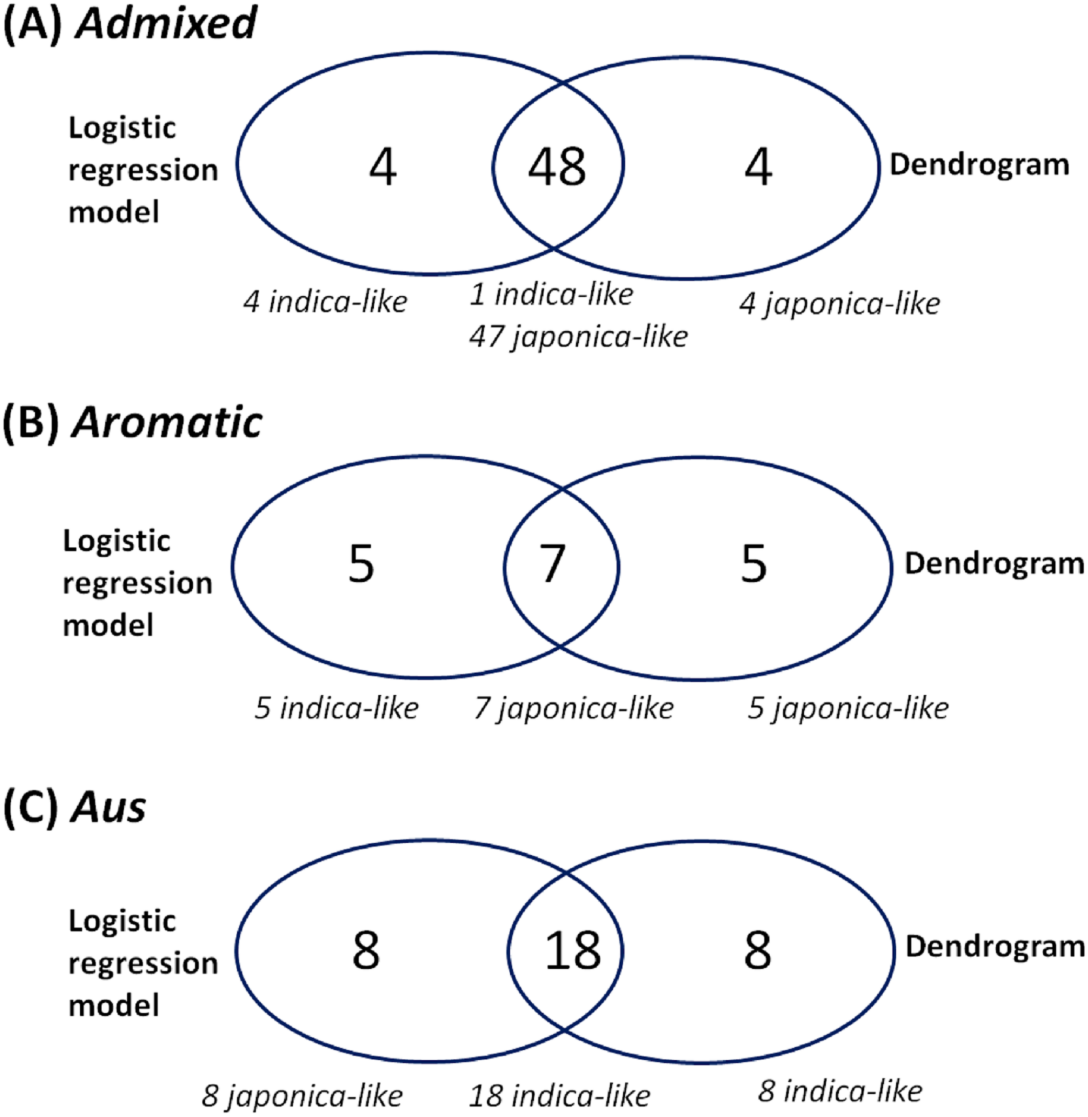
Comparison between the LRM-based classification and the dendrogram-based classification for each minor subpopulation group. (A) Comparison between the LRM-based classification and dendrogram-based classification for the *admixed* group, (B) comparison between the LRM-based classification and dendrogram-based classification for the *aromatic* group, (C) comparison between the LRM-based classification and dendrogram-based classification for the *aus* group.

Because of missing phenotypic records, the size of each minor subpopulation group was reduced from 62 to 52 for the *admixed* group, 14 to 12 for the *aromatic* group and 57 to 26 for the *aus* group. Figure 3 shows that Eq. (3) split each subpopulation group into *indica* and *japonica* in ratios of 5:47 for the *admixed* group, 5:7 for the *aromatic* group and 18:8 for the *aus* group, respectively. Meanwhile, the dendrogram drawn with the whole accessions (413) shows two major clades (upper and lower), in which *temperate japonica*, *tropical japonica* and *aromatic* formed accurately separate groups within the upper clade (hereafter called *japonica*-varietal clade), while *indica* and *aus* formed accurately separate groups within the lower clade (hereafter called *indica*-varietal clade). The *admixed* accessions were spread across all subpopulation groups (Fig. S2). Figure 4 shows three Venn diagrams, each of which represents comparison between the LRM-based and dendrogram-based classifications for each minor subpopulation group; the agreements were 92.3% (48/52) in the *admixed* group, 58.3% (7/12) in the *aromatic* group and 69.2% (18/26) in the *aus* group.

## Discussion

The *indica* and *japonica* in *Oryza sativa* can be classified based on phenotypic observations by humans. However, the classification by humans can be subjective so that the classification results could sometimes be biased by an observer’s perception. Meanwhile, the *indica*/*japonica* classification by the LRM is thoroughly systematic and quantitative. In this aspect, the *indica*/*japonica* classification qualities by the LRM are expected to be comparable to or better than the qualities made by humans. In fact, the customized LRM (Eq. (3)) achieved the *indica*/*japonica* classification accuracy of 100% given the 280 *indica*/*japonica* accessions.

Table 1 and Fig. 1 suggest that the more predictor variables there are, the stronger the *indica*/*japonica* separation power (AUC). This implies that the variation in a single trait is narrowly distinct between *indica* and *japonica* perhaps due to intensive genetic admixture by breeding over history (Zhao et al., 2010; Xu et al., 2012), and that the variation given multiple traits is substantially distinct between *indica* and *japonica* because multiple layers of the narrow effects collectively magnify differences between *indica* and *japonica*. In this study, the fully accurate *indica*/*japonica* separation power (AUC = 1) of the LRM was achieved with a set of seven phenotypic variables (Table 1 and Fig. 1). The *H*-statistic value of nearly zero for every phenotypic variable indicates that the *indica*/*japonica* separation power was not overestimated by unexpected synergetic effects between the phenotypic variables.

Table 2 shows that the panicle-related traits, straighthead susceptibility and blast resistance frequently appeared across all sets. This may be related to previous knowledge that the variations in panicle characteristics, straighthead susceptibility and blast resistance are strongly associated with the *indica* and *japonica* differentiation: panicles are long and sparse in *indica* but short and dense in *japonica* (Bai et al., 2016); straighthead resistance and blast resistance are greater in *indica* than *japonica* (Yan et al., 2005; Jia et al., 2011).

The customized LRM (Eq. (3)) was applied to each minor subpopulation group (*aromatic*, *aus*, *admixed*). Applying Eq. (3) to the *aromatic* and *aus* groups aimed to see how well the resulting classifications reflect their known evolutionary relationships with both *indica* and *japonica*: *aromatic* is intermediate but narrowly closer to *japonica*; *aus* is distinct from but closely related to *indica* (Garris et al., 2005; Thomson et al., 2007; Kovach et al., 2009; Huang et al., 2012; Schatz et al., 2014; Civan et al., 2015; McCouch et al., 2016; Chin et al., 2017). Regarding the *aromatic* accessions, the dendrogram-based classification formed the *aromatic* group distantly from the *japonica* group in the *japonica*-varietal clade, which is consistent with the previous knowledge that *aromatic* is narrowly close to *japonica* between *indica* and *japonica*. The agreement between the dendrogram-based and LRM-based classifications was 58.3% (7/12). The low agreement may be related to the subtle phenotypic similarity between *aromatic* and *japonica* (Garris et al., 2005). Regarding the *aus* accessions,** the dendrogram-based classification assigned all of them to the *aus* group in the *indica*-varietal clade, which was consistent with the previous knowledge that *aus* and *indica* are distinct within a close evolutionary relationship. The agreement of 69.2% (18/26) between the dendrogram-based and LRM-based classifications suggests that phenotypic variation between the *aus* and *japonica* groups overlaps to some extent. Perhaps, it may be because the sub-speciation of *aus* from *indica*, occurred in a geographically isolated area (Bangladesh, India) under short growing seasons and upland conditions, might have confounded its phenotypic characteristics (Garris et al., 2005; Londo et al., 2006). Regarding the *admixed* accessions, the dendrogram-based classification dispersed them across *indica*- and *japonica*-varietal clades. This indicated that the *admixed* group covered a wide spectrum of genomic properties. The *admixed* group was a collection of accessions with uncertain subpopulation membership due to intensive inter-subpopulation mating. This allows us to deduce that the *admixed* accessions may have a high potential to bridge between *indica* and *japonica*. The agreement of 92.3% (48/52) between the dendrogram-based and LRM-based classifications shows reliable classification ability of the LRM given *Oryza sativa* accessions with uncertain subpopulation membership.

## Conclusion

This study showed that the *indica*/*japonica* classification based on phenotypes can be analyzed using the LRM. A set of phenotypes that can collectively generate the fully accurate *indica*/*japonica* separation power can be used for researching *indica*/*japonica* differentiation. For example, if a study aims to detect quantitative trait loci (QTL) associated with the *indica*/*japonica* differentiation, each phenotype that contributes to the fully accurate *indica*/*japonica* separation power can be used for genome-wide association studies (GWAS). Furthermore, research on the *indica*/*japonica* differentiation in *Oryza sativa* may benefit from the variation of genes responsible for the phenotypes that contribute to the fully accurate *indica*/*japonica* separation power.

##  Supplemental Information

10.7717/peerj.7259/supp-1 Figure S1 Dendrogram that represents a classification of 280 indica/japoinca accessions

10.7717/peerj.7259/supp-2 Figure S2 Dendrogram that represents a classification of 413 admixed/aromatic/aus/indica/japonica accessions

## Additional Information and Declarations

### Data Availability

The following information was supplied regarding data availability: Data is available at GitHub: https://github.com/bongsongkim/logit.regression.rice.
