# Supplementary figures and images for: Classifying Oryza sativa accessions into Indica and Japonica using logistic regression model with phenotypic data
Source: PeerJ. 2019 Nov 7;7:e7259. doi: 10.7717/peerj.7259 (PMC6842562; doi:10.7717/peerj.7259)

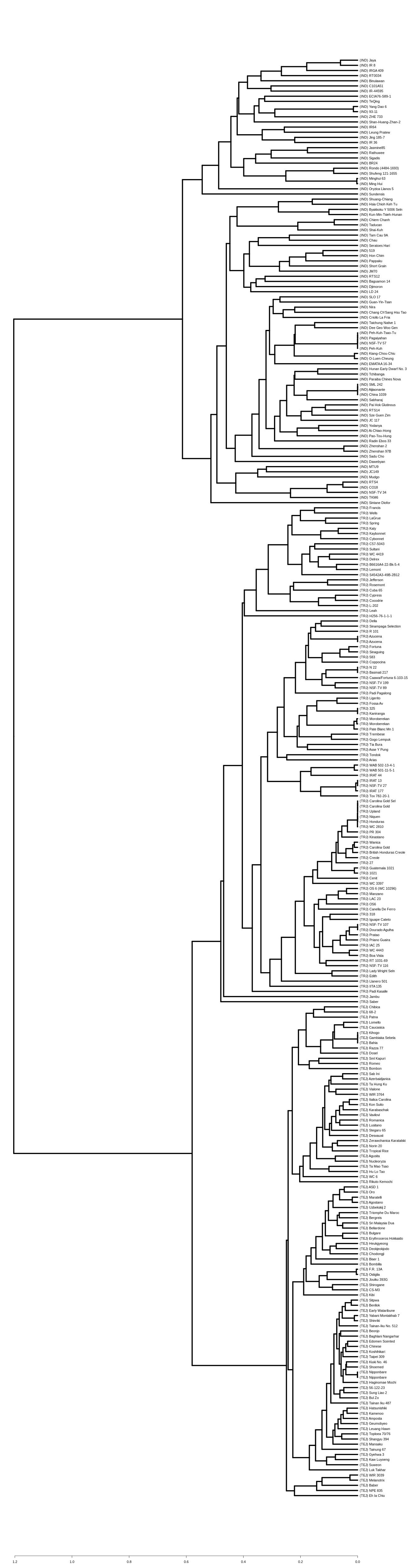

Supplement: Figure S1 [file peerj-07-7259-s001.jpg]

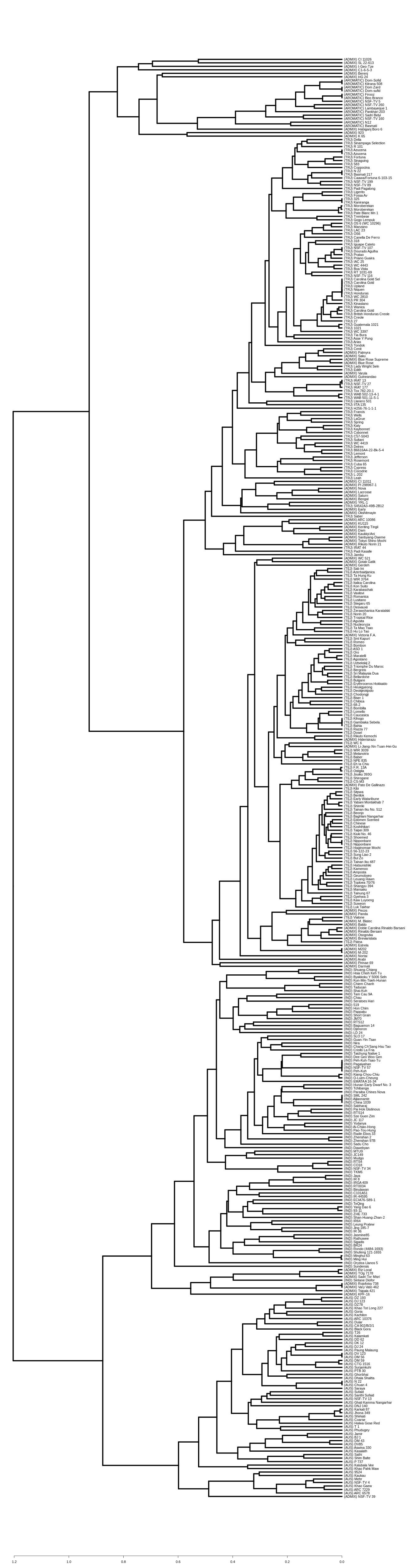

Supplement: Figure S2 [file peerj-07-7259-s002.jpg]
